# The Quantified Level of Circulating Prostate Stem Cell Antigen mRNA relative to GAPDH Level Is a Clinically Significant Indictor for Predicting Biochemical Recurrence in Prostate Cancer Patients after Radical Prostatectomy

**DOI:** 10.1155/2015/292454

**Published:** 2015-10-07

**Authors:** Sung Han Kim, Weon Seo Park, Sang Jin Lee, Moon Kyung Choi, Seung Min Yeon, Jeong Nam Joo, Ara Ko, Eun Sik Lee, Jae Young Joung, Ho Kyung Seo, Jinsoo Chung, Kang Hyun Lee

**Affiliations:** ^1^Department of Urology, Center for Prostate Cancer, Research Institute and Hospital of National Cancer Center, Goyang 410-769, Republic of Korea; ^2^Department of Pathology, Center for Prostate Cancer, Research Institute and Hospital of National Cancer Center, Goyang 410-769, Republic of Korea; ^3^Genitourinary Cancer Branch, Center for Prostate Cancer, Research Institute and Hospital of National Cancer Center, Goyang 410-769, Republic of Korea; ^4^Division of Specific Organ Cancer, Center for Prostate Cancer, Research Institute and Hospital of National Cancer Center, Goyang 410-769, Republic of Korea; ^5^Biometric Research Branch, National Cancer Center, Goyang 410-769, Republic of Korea; ^6^Department of Urology, Seoul National University Hospital of Medicine, Seoul 110-744, Republic of Korea

## Abstract

The study quantified the relative absolute PSCA level in relation to the glyceraldehyde 3-phosphate dehydrogenase (GAPDH) level in the peripheral blood of 478 hormone-naive prostate cancer (PC) patients who underwent radical prostatectomy from 2005 to 2012 and evaluated its prognostic significance as a risk factor for predicting biochemical recurrence (BCR), compared to known parameters. Nested real-time polymerase chain reaction (RT-PCR) and gel electrophoresis detected PSCA levels and measured the PSCA/GAPDH ratio. Clinicopathological data from the institutional database were examined to determine the adequate cut-off level to predict postoperative BCR. A total of 110 patients had a positive PSCA result (23.0%) via RT-PCR (mean blood ratio 1.1 ± 0.4). The BCR was significantly higher in the PSCA-positive detection group (*p* = 0.009). A multivariate model was created to show that a PSCA/GAPDH ratio between 1.0 and 1.5 (HR 12.722), clinical T2c stage (HR 0.104), preoperative PSA (HR 1.225), extraprostatic capsule extension (HR 0.006), lymph node dissection (HR 16.437), and positive resection margin (HR 27.453) were significant predictive factors for BCR (*p* < 0.05). The study showed successful quantification of PSCA with its significance for BCR-related risk factor; however, further studies are needed to confirm its clinical predictive value.

## 1. Introduction

Prostate cancer (PC) has become the most frequent malignancy in men and causes the second highest number of cancer-related deaths. Half of all patients have metastatic disease at the initial diagnosis, and nearly half of the remainder who present with an initially localized disease will develop subsequent metastasis despite appropriate treatments [1]. To date, there have been no completely accurate diagnostic tools for controlling advanced disease states or predicting the early progression of PC during follow-ups; therefore, many clinicians are searching for new tumor markers or other methods to improve their detection rate.

Recent research has emphasized circulating tumor cells (CTCs), detected in the blood or lymphatic fluid from the primary PC lesion, as a potentially predictive micrometastatic tumor cell. This type of cell may metastasize to other organ sites when adequate conditions for survival are met in the secondary site. The CTC count is thus considered important for predicting PC progression, determining likely treatment outcomes, and choosing early preventive measures.

In particular, prostate stem cell antigen (PSCA) has recently come under scrutiny as a potential CTC marker. The antigen is found at low expression levels in the normal state but increases when the prostate's condition becomes malignant and during progression from an early to an advanced PC state [2, 3]. The reverse transcriptase polymerase chain reaction (RT-PCR) has successfully quantified PSCA [4], prompting many other researchers to evaluate the benefits of PSCA as a diagnostic tool and explore its use in the treatment and prevention of PC in clinical and animal models [5–7].

Although this basic nested RT-PCR method for quantification of PSCA has not proven to be particularly precise upon repeated studies [8], a group from China recently used the RT-PCR method to show that circulating levels of PSCA correlated with androgen-independent progression in advanced PC [9]. Joung et al., from our institution, also showed the objective utility of using nested RT-PCR to quantify peripheral blood levels of PSCA mRNA to find several important genotypes and to predict the likelihood of biochemical recurrence (BCR) [10, 11].

Therefore, this study aimed to quantify absolute PSCA level in the peripheral blood of PC patients who underwent radical prostatectomy and relate it to a GADPH reference level (PSCA/GAPDH ratio) using RT-PCR. The study further aimed to determine PSCA's reliability as a tumor biomarker in comparison with other known clinicopathological prognostic parameters of BCR.

## 2. Materials and Methods

This study was approved by the Institutional Review Board (IRB number NCCNCS 05-049) with written consent from the participants. It was conducted according to the principles expressed in the Declaration of Helsinki.

### 2.1. Patient Selection and Blood Samples

From February 2005 to December 2012, 478 PC patients who underwent radical prostatectomy and standard pelvic lymph node dissection at the Center for Prostate Cancer, National Cancer Center, were prospectively selected for screening and possible inclusion into the study with a mean follow-up time of 42.1 ± 25.30 months. All cases were pathologically confirmed as adenocarcinomas, based on current World Health Organization criteria and Gleason grade by a single experienced uropathologist (Professor WSP, MD, PhD). Other clinicopathologic data were prospectively recorded in the Prostate Cancer Center database. No postoperative adjuvant hormonal or radiotherapies were performed until biochemical recurrence (BCR) developed, defined as a postoperative serum PSA elevation of >0.2 ng/mL assessed on two occasions, following a prior decrease to nondetectable levels. The first PSA value of 0.2 ng/mL or greater was used to define the time of recurrence.

Researchers collected preoperative blood samples and performed RT-PCR to detect PSCA. A total of 135 (28.2%) cases tested positive for PSCA. Exclusion criteria included refusal to participate in the study; missing preoperative clinicopathological data; inadequate volume of PSCA cDNA for RT-PCR; previous history of hormone therapy or chemotherapy or radiation therapy; history of invasive prostate treatment; postoperative loss to follow-up; or short follow-up less than 1 year. After excluding 25 patients based on these criteria, 110 (23.0%) patients with PSCA-positive preoperative blood samples were ultimately enrolled.

### 2.2. Prospectively Collected mRNA and cDNA Extraction and Reverse Transcription

Blood samples of 20 mL were obtained from patients just before the RP operation. Samples were delivered to the laboratory within an hour and nucleated cell fractions were isolated from 5 mL whole blood samples using Percoll Gradient Centrifugation (Amersham Biosciences, Uppsala, Sweden). Then, total RNA, extracted from nucleated cells, was converted to cDNA by reverse transcriptase and analyzed by PCR and nested PCR, as described in previous reports [10, 11]. After RT-PCR assay sensitivity in cultured LNCaP cells was completed, assay products were evaluated to determine PSCA positivity. A 2% agarose gel was used for electrophoresis, and an ultraviolet transilluminator (UV-T) was used for visualization. All 110 PSCA-positive samples were collected and stored at −70°C.

### 2.3. PSCA Quantification

The mRNA obtained from 110 PSCA-positive samples was converted to cDNA, and PCR/nested PCR reactions from the prepared cDNA were utilized to calculate the amount of PSCA mRNA. The PSCA transcript quantities were normalized with an internal glyceraldehyde 3-phosphate dehydrogenase (GAPDH) mRNA control.

The PCR-reaction mixture for quantified analysis included 5 L of cDNA, 10 *μ*L of buffer (Go Taq), 0.25 *μ*L of DNA Polymerase (Promega Go Taq, Promega, WI), 4 *μ*L of dNTP 2.5 mM mixture (TaKaRa, Japan), ≤50 *μ*L of distilled water, and 1 *μ*L of each primer.

All primers used in this study were synthesized by Bioneer (Daejeon, Korea). The primers specific for human PSCA were 5′-CCC TGC AGC CAG GCA CT-3′ and 5′-AGG CCA ACT GCG CG AT-5′. Primers for human GAPDH were 5′-TGG TCA CCA GGG CTG CTT TTA-3′ and 5′-TCC TGG AAG ATG GTG ATG GGA TTT-3′. For nested PCR for PSCA detection, primers were 5′-CAC TGC CCT GCT GTG CTA CT-3′ and 5′-CGC GGT CCA GCA CTG CTC CC-3′. For the internal control to assess RNA integrity, GAPDH was amplified. PCR reactions were performed in a total volume of 50 *μ*L containing 1 *μ*L of RT product, 5 *μ*L of 10x PCR-reaction buffer (200 mmol/L Tris HCl, pH 8.4, 500 mmol/L KCl), 0.1 *μ*mol/L sense and 0.1 *μ*mol/L antisense primer, 0.2 mmol/L of each dNTP, and 1 U Taq-DNA polymerase.

Amplification of 5 *μ*L cDNA was performed using a Tube Controlled Thermal Cycler (MJ Research, Waltham, MN, USA) with the following reaction profile: 2 min of initial denaturation at 95°C followed by single cycle of denaturation at 95°C for 30 s; 30 s of annealing at 55°C followed by 35 cycles at 94°C for 30 s, 55°C for 1 min, and 72°C for 1 min; and a final extension at 72°C for 5 min. For nested PCR, 5 *μ*L of PCR product was amplified with the same reagents and with nested primers for an additional 35 cycles. LNCaP cells were cultured, and RT-PCR assay sensitivity was determined, as described in a previous report [10, 11]. The sensitivity of LNCaP cells and other markers were all analyzed to confirm the amount of PSCA transcripts using GADPH as a reference.

Quantification was performed on 1% agarose gel treated with 0.5 *μ*g/mL ethidium bromide stain. Then, the UV-T was used to visualize the fluorescent expression of PSCA and GAPDH, and the relative amount of PSCA transcripts was determined.

### 2.4. Statistical Analysis

The chi-squared and Fisher's exact tests were used to evaluate the correlation of PSCA titer and PSCA/GAPDH ratio with clinicopathological factors. Student's* t*-test was used to compare differences in BCR. In this study, disease progression was defined as BCR after prostatectomy. Multivariate survival analysis was evaluated using Cox's proportional hazard models to identify independent prognostic factors of BCR. Two-sided *p* values < 0.05 using STATA (release 9.2, STATA Inc., TX, USA) were considered statistically significant.

## 3. Results

**Figure 1:**
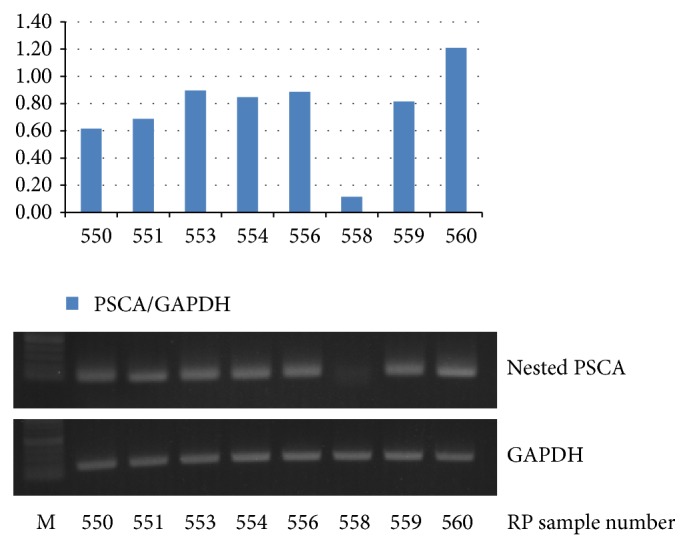
Detection of PSCA and GAPDH positivity using gel electrophoresis with ultraviolet transilluminator.

The clinicopathologic characteristics and demographics of the 110 positive PSCA patients are described in Table 1. Using nested RT-PCR, a mean PSCA titer of 9531.2 ± 4272.5 was detected, and a relative mean PSCA/GADPH ratio of 1.1 ± 0.4 was detected via gel electrophoresis and UV-T (Table 1, Figure 1).

**Figure 2:**
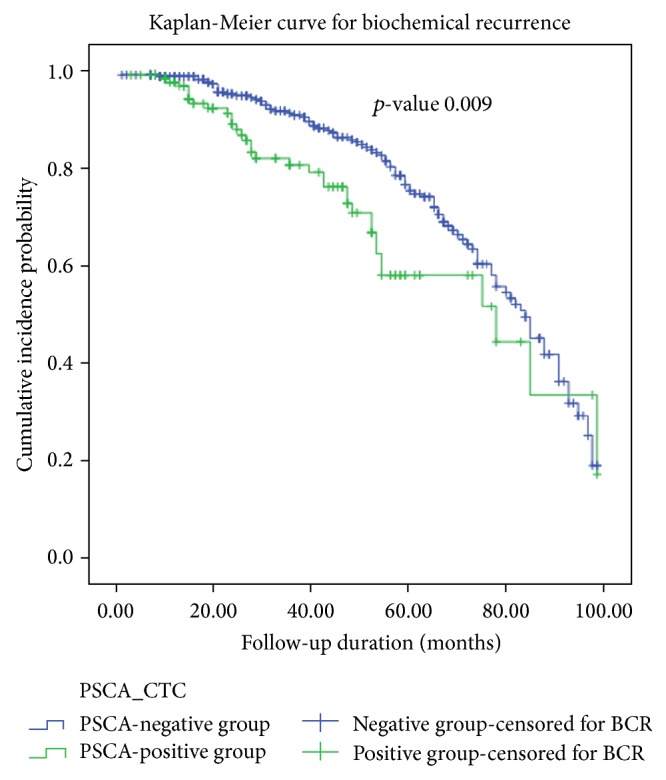
Kaplan-Meier curve for biochemical recurrence-free survival between PSCA-negative and PSCA-positive groups.

The correlation analysis of PSCA titers and known clinicopathologic prognostic parameters showed that clinical (odds ratio [OR] 15.049) and pathologic T stage presence (OR 8.431), a positive resection margin (OR 9.545), and apical tumor involvement (OR 13.291) were significantly correlated with PSCA titer, and BCR (OR 8.091) and perineural invasion (OR 8.233) were significantly correlated with the PSCA/GAPDH ratio (*p* < 0.05, Table 2). The BCR-free survival curve showed that PSCA-positive group had higher incidence of BCR than the negative group (*p* = 0.009, Figure 2).

To determine the PSCA/GAPDH ratio as a predictive prognostic factor for BCR, univariate and multivariate analyses were performed. The univariate analysis showed that the preoperative PSA, PSCA absolute titer, PSCA/GAPDH ratio, pathologic Gleason score sum, clinical T stage, positive resection margin, lymphovascular invasion, perineural invasion, extraprostatic capsule extension, seminal vesicle invasion, lymph nodal dissection, and pathologic T stage were significantly different (*p* < 0.05, data not shown). The multivariate Cox regression model showed that a PSCA/GAPDH ratio of 1.0–1.5 (HR 12.722, 95% CI 1.737–843.933), a clinical T2c stage (HR 0.104, 95% CI 0.018–0.621), preoperative PSA levels (HR 1.225, 95% CI 1.068–1.406), extraprostatic capsule extension (HR 0.006, 95% CI 0.001–0.271), lymph node dissection (HR 16.437, 95% CI 1.024–263.859), and positive resection margin (HR 27.453, 95% CI 3.5–215.318) remained significant risk factors for BCR (*p* < 0.05, Table 3).

## 4. Discussion

A blood-based examination of PSA has historically been the most popular diagnostic screening tool for predicting PC risk and outcome; however, it is difficult to accurately predict the progression of advanced and localized PC even after adequate treatment based solely on PSA results, because the hormone's values can fluctuate up to 20–30% based on biological and environmental factors [12]. Therefore, many clinicians and researchers have suggested alternative prognostic measurements and markers such as derivatives of PSA kinetics, other imaging modalities, or novel tumor biomarkers to supplement or fully replace PSA and better predict PC prognosis and progression [13].

Recently, focus has shifted to micrometastatic CTC biomarkers in peripheral blood [13–15]. In particular, PSCA has received recent scrutiny as a biomarker because it can accurately define the correlations between PC prognosis, diagnosis, and preventive measures [16, 17]. Highly overexpressed in human PC but with only limited expression in normal tissues, PSCA is a cell-surface antigen that belongs to the Ly-6/Thy-1 family of glycosylphosphatidylinositol-anchored proteins. The function of PSCA in normal and tumor contexts is not perfectly known; however, the Thy-1 family is known to play a role in T cell activation, proliferation, stem cell survival, and cytokine and growth factor responses. Furthermore, PSCA has already been proven to play a specific role in and have a strong relationship with PC development, because the Ly-6 family is associated with carcinogenesis, cellular activation, and cell adhesion of tumor cells [2, 18, 19].

Our institution has been conducting prospective studies to find potential tumor biomarkers in the peripheral blood of Korean PC patients since 2005, and we have further reinforced the potential of PSCA as a potential PC tumor biomarker [10, 11]. The marker has already been proven to have a significant correlation with prognostic parameters of BCR, and the genetic specificity of haplotype increased the risk of PC prevalence. As our previous studies qualitatively defined PSCA as a potential PC biomarker after radical prostatectomy, we conducted this study to definitively quantify the clinical usefulness of PSCA titer in blood samples by more accurately defining the PSCA/GAPDH ratio as a significant prognostic tumor biomarker for BCR.

In this study, 28.2% (*N* = 135) of peripheral blood samples from 478 patients' blood samples were positive for PSCA, and 23.0% (*N* = 110) of samples were ultimately include. The included patients had significantly lower rates of BCR-free survival compared to others in the Kaplan-Meier plot, assessed via the log rank test (*p* = 0.009, Figure 2). The quantified PSCA/GAPDH ratio was analyzed in both correlation and multivariate models to determine its potential predictive risk factor for BCR (Tables 2 and 3). The analyses showed that the PSCA/GAPDH ratio was related to BCR and its stratified value of 1.0–1.5 (HR 12.722) was found significant for predicting BCR after radical prostatectomy (*p* < 0.05) with other significant clinical T2c stage (HR 0.104), lymph node dissection (HR 16.437), positive resection margin (HR 27.453), extraprostatic capsule extension (HR 0.006), and preoperative PSA (HR 1.225), which have been already shown in previous studies (*p* < 0.05, Table 3) [20–22].

Background analyses were performed to find an appropriate discriminating cut-off value and its clinical value in predicting the BCR. An arbitrary cut-off value of 1.2 around a median value between 1.0 and 1.5 showed a statistical discriminating power of BCR-free survival curve in the Kaplan-Meier curve with log rank test (*p* = 0.034, not shown in figure). However, this value was not finally proven to be valid for determining the statistical significance because external validation could not be performed and internal validation failed due to the limited number. Although this study has limited generalizability in defining a specific value of PSCA/GAPDH ratio due to the limited number of samples and lack of internal and external validations, this study is clinically significant because, for the first time, a study successfully suggested that quantified PSCA with a significant range value could be a potential prognostic factor for BCR and an alternative follow-up biomarker to PSA after radical prostatectomy.

Most previous PSCA studies have been based on Western patients or animal modeling studies, and only a few Asian studies have reported PSCA as a tumor biomarker strongly associated with clinicopathological parameters such as the Gleason score, pathologic stage, seminal vesicle invasion, extraprostatic capsule extension, and metastasis [4, 9, 23]. Their findings of PSCA correlation to disease invasiveness and progression were similar to our findings (Table 2). Unfortunately, however, none of those previous studies were prospectively designed, involved large numbers of samples from PC patients underwent radical prostatectomy, or involved long-term follow-up and they did not investigate PSCA's genetic, qualitative, and quantitative aspects, as was done in the present study and our previous studies [10, 11]. Thus, no definitive conclusions can yet be made, although results remain encouraging. Additionally, the subanalytic correlation study of stratified PSA, Gleason score, and pathologic T stage showed that none of the significant stratified groups of PSA, Gleason score, and pathologic T stage were significantly related to the PSCA/GAPDH ratio (*p* > 0.05, data not shown). The PSCA/GAPDH ratio would thereby be an independent biomarker for these three markers.

The limitations of this study included the retrospective nature of the quantitative analysis, the inaccuracy of nested RT-PCR using UV-T for quantification of PSCA positivity, and no internal and external validation of our arbitrary PSCA/GAPDH cut-off value. Further larger analyses with more sensitive and accurate modalities to detect the quantified PSCA in blood samples, such as recently introduced detection equipment using nanotechnologies, are required. Additionally, longer-term follow-up is necessary to fully evaluate the prognostic value of PSCA including overall survival and progression-free survival to hormone refractory state in PC.

## 5. Conclusion

The study showed that a patient's quantified PSCA ratio to GAPDH (PSCA/GAPDH ratio) was significantly correlated with BCR and if its value was between 1.0 and 1.5, it was a significant risk factor for BCR. This suggested the possibilities of using PSCA/GAPDH ratio as an alternative tool for prognosis prediction than what has been examined to date. Further studies with more accurate analytic methods should be considered to detect the titers of PSCA in the peripheral blood of PC patients as an alternative to current PSA tests.

## Figures and Tables

**Table 1:** Patients' clinicopathologic characteristics (*N* = 110).

Parameters	*N* (%) or mean ± SD
Preoperative PSA (ng/mL)	11.7 ± 10.3
Prostate volume (mL)	35.5 ± 16.1
PSCA absolute titer	9531.2 ± 4272.5
>15,000	9 (8.2)
10,1001–1,5000	38 (34.5)
5001–10,000	51 (46.4)
<5000	12 (10.9)
PSCA/GAPDH ratio	1.1 ± 0.4
>1.5	11 (10.0)
1.1–1.5	56 (50.9)
0.5–1.0	35 (31.8)
>0.5	8 (7.3)
Gleason score sum	6.6 ± 0.8
Clinical stage T1	36 (32.7)
T2	56 (50.9)
T3	16 (14.6)
N1	2 (1.8)
Pathologic Gleason score sum	7.0 ± 0.9
Pathologic prostate volume (mL)	33.8 ± 15.2
Pathologic stage ≤T2b	63 (57.3)
≥T2c	47 (42.7)
N (+)	1 (0.9)
M1	4 (3.6)
Biochemical recurrence	24 (21.8)
Median time to biochemical recurrence (months)	7.0 (3–46)
Median follow-up duration (months)	42.1 ± 25.0

**Table 2:** Correlation of prostate stem cell antigen titer with known clinicopathological prognostic parameters (*B* = 110).

Parameters	Correlation with PSCA absolute titer (*p* value)	Correlation with PSCA/GAPDH (*p* value)
Age (years)	0.601	0.685
Preoperative PSA (ng/mL)	0.457	0.639
Preoperative-free PSA (ng/DL)	0.357	0.458
Prostate size (mL)	0.458	0.385
Gleason score sum at biopsy	0.508	0.741
Clinical T stage (T1, T2, ≥T3)	0.010 (OR 15.049)	0.485
Positive resection margin	0.018 (OR 9.545)	0.125
Pathologic Gleason score sum	0.630	0.571
Pathologic T stage (≤T2b or ≥T2c)	0.033 (OR 8.431)	0.187
Pathologic N or M stage	0.234	0.259
Apex involvement	0.003 (OR 13.291)	0.643
Lymphovascular invasion	0.643	0.053 (OR 7.934)
Perineural invasion	0.059 (OR 7.419)	0.039 (OR 8.233)
Extracapsular extension	0.051 (OR 7.658)	0.108
Seminal vesicle invasion	0.810	0.950
Biochemical recurrence	0.681	0.032 (OR 8.091)

OR: odds ratio; HGPIN: high grade prostatic intraepithelial neoplasm; PSA: prostatic specific antigen.

**Table 3:** Multivariate analysis of BCR-related risk factors using stratified PSCA/GAPDH ratio into three subgroups.

	*p* value	Hazard ratio	95.0% confidence interval	
			Lower limit	Upper limit
Lymphovascular invasion	0.072	0.059	0.003	1.293
Perineural invasion	0.721	0.612	0.042	9.024
Extraprostatic capsule extension	0.008	0.006	0.001	0.271
Seminal vesicle invasion	0.589	1.464	0.367	5.838
Lymph node dissection	0.048	16.437	1.024	263.859
Resection margin negative	0.002	27.453	3.500	215.318
Neurovascular saving	0.110	4.217	0.723	24.603
Clinical T1	0.006			
cT2a, T2b	0.165	0.077	0.002	2.864
cT2c	0.013	0.104	0.018	0.621
≥cT3	0.083	14.700	0.706	306.063
Pathologic Gleason score	0.801	1.256	0.213	7.397
PSCA/GAPDH >1.5	0.052			
1.0–1.5	0.016	12.722	1.598	101.286
<1.0	0.135	5.403	0.590	49.499
Preoperative PSA	0.004	1.225	1.068	1.406
Pathologic ≤T2b	0.494	0.403	0.030	5.468

## Abbreviations

cDNA: Complementary DNA
dNTP: Deoxynucleotide triphosphate
LNCaP: Oncological cell line
mRNA: Messenger RNA.
